# An Insect Worth Millions

**Published:** 1891-12

**Authors:** 


### AN INSECT WORTH MILLIONS.

The cottony cushion scale, says the *Indiana Farmer*, has been for years the greatest enemy to the orange grower of California. It was brought there from Australia in 1868, on trees and shrubs imported from that country, and spread and multiplied till it promised soon to devastate the entire orange growing regions of the State. A fruit grower near San Francisco, under the belief that there must be a parasite for the insect in the country from which it came, went to Australia, and after due investigation, made the expected discovery. It was the lady-bird, the *Vedalia cardinalis*, he found to be the principal enemy of the cottony cushion scale, and he captured and shipped several colonies of beetles and their larvæ to California.

This was the fall of 1888. According to bulletin No. 54 of the California State Board of Horticulture, so rapid was their increase that by December 1, 1889, the work of exterminating the cottony cushion scale was practically accomplished. The money value of this *Vedalia* to the orange growers of the State has been incalculable. The saving of the orchards already infested, the protecting of the others that were sure to be blighted by this terrible curse, to say nothing of perpetuating an industry that it seems will be the king of all horticultural pursuits, is simply grand, and cannot be estimated in the usual dollar and cent test.

